# Covid-19 psychological pressures, depression and FOMO: the mediating role of online social support and emotional regulation

**DOI:** 10.1186/s40359-024-01610-2

**Published:** 2024-03-02

**Authors:** Yuting Dong, Min Chen, Zhigang Wu, Zilin Zhang

**Affiliations:** 1 School of Education, Northwest Normal University, Lanzhou, 730070 Gansu China; 2 School of Energy and Mechanical Engineering, Jiangxi University of Science and Technology, Nanchang, 330000 Jiangxi China; 3 Historic Building Division, China Construction First Division Group Huajiang Construction Co., Ltd, Jingdezhen, 333099 Jiangxi China

**Keywords:** Covid-19 psychological pressures, Depression, FoMO, Mediation, Online social support, Emotional regulation

## Abstract

**Background:**

The spread of the coronavirus has led to significant anxiety among university students, resulting in various mental health problems that could potentially impact their academic performance.

**Method:**

To examine the mediating role of emotional regulation and online social support in the relationships between COVID-19 psychological pressures, depression, and the fear of missing out (FoMO) among young adult university students, a cross-sectional research design was employed using an online survey. The sample consisted of 521 full-time university students from China, currently enrolled in undergraduate and postgraduate programs.

**Results:**

Findings revealed that more than half (55.09%, *n*=287) of the university students experienced COVID-19 psychological pressures. These pressures directly contributed to increased levels of depression (*β* = 0.339, *p* < .001) and fear of missing out (*β* = 0.236, *p* < .001). Moreover, online social support and emotional regulation exhibited partial mediating effects on the association between COVID-19 psychological pressures, depression, and the fear of missing out. The results indicated that COVID-19 psychological pressures were linked to higher levels of depressive symptoms and a greater fear of missing out among university students.

**Conclusions:**

However, the provision of timely and adequate online social support, as well as the implementation of emotional regulation strategies, mitigated the negative effects of the pandemic on students' social and emotional well-being. Consequently, this led to reduced levels of depression and fear of missing out.

## Background

A novel coronavirus pneumonia disease, COVID-19, rapidly spread throughout China in early 2020 [1]. The outbreak was initially identified at the end of December 2019 when several unexplained pneumonia cases were linked to an undetected exposure at the fish market in Wuhan, Hubei Province, China [2]. On March 11, 2020, the World Health Organization announced the identification of a new strain of the coronavirus family, named SARS-CoV-2, which was responsible for the COVID-19 outbreak [3, 4]. Due to its high contagion rate and lack of a definitive treatment, COVID-19 rapidly spread worldwide and had a significant impact on a large number of individuals [5]. The extensive contamination and strain on healthcare systems globally led to heightened anxiety among individuals. The coronavirus outbreak sparked significant concern worldwide, resulting in a range of psychological and mental health issues [6–9].

Consequently, a multitude of individuals have experienced a range of mental health challenges including stress, anxiety, depression, poor sleep quality, mood swings, and high levels of post-traumatic stress disorder symptoms [10–13]. Among the various mental health issues that have emerged as direct consequences of the COVID-19 pandemic, stress, pressures, and anxiety have been particularly concerning, especially among young adults who harbor fears of contagion during this global crisis [14–17].

Stress and anxiety represent significant concerns for psychologists, psychiatrists, and behavioral scientists worldwide [18]. Stress has been defined as a process wherein environmental pressures surpass an individual's capacity to adapt, resulting in psychological and biochemical changes that may predispose individuals to disease [19]. Folkman [20] argues that stress is an adaptive process that elicits varied reactions when the internal and external environments are out of balance. Richard Lazarus co-developed the Transactional Model of Stress and Coping (TSC) in collaboration with Susan Folkman. Lazarus and Folkman's (1984) model has had a significant impact and continues to be the foundation of research on psychological stress and coping in other disciplines (Biggs, Brough & Drummond, 2017; Christensen et al., 2004).

According to the TSC (Folkman, 2008; Lazarus, 2006; Lazarus & Folkman, 1984), two prototypical states emerge from cognitive assessments of the significance of a situation and an individual's capacity to react to it: challenge and threat. Primary appraisal, as used in the TSC, pertains to the evaluation of whether a given situation is benign or distressing. Benign circumstances are perceived as necessitating no deliberate action to bring about a favorable result, whereas distressing situations demand specific actions. Stressful circumstances can be classified as either confronting or threatening in nature. Situations that are perceived as presenting opportunities for improvement, mastery, and gain are considered challenging (e.g., excelling in examinations). Dangerous circumstances are those that are perceived as having the potential to cause damage or loss (e.g., failing an examination) (Ben-Zur, 2019; Putwain, et al., 2021).

On the other hand, secondary appraisal of one's capacity to cope with stressful circumstances ascertains the degree of threat or challenges perceived. The threat stems from individuals lacking the requisite capabilities to manage the situation (e.g., despite being aware of the significance of the exam, I maintain confidence in my own capabilities). The challenge arises from the perception that one possesses the requisite resources (e.g., despite being cognizant of the significance of the exam, I maintain a negative outlook regarding my ability to pass it). While primary appraisal may suggest that assessments of the significance or relevance of a situation come before evaluations of coping, this is not always the case. For example, secondary appraisals can ascertain the initial significance of a given circumstance (Ben-Zur, 2019; Blascovich & Mendes, 2010; Jamieson, 2017).

Therefore, the TSC model underscores the person–environment transaction, highlighting the influential role of individual appraisal processes in shaping stress responses. When confronted with stressors, individuals engage in primary appraisal, evaluating the stressors' relevance, followed by secondary appraisal, assessing personal resources to cope. These appraisals guide the selection of coping strategies, which, in turn, influence immediate stress responses and long-term outcomes in terms of health, psychological well-being, and social functioning. The model, presented in a linear section for simplicity, acknowledges its dynamic and recursive nature, with footnotes indicating the parallelism of short- and long-term effects (Lazarus and Folkman, 1987). Lazarus and colleagues later refined their theory, introducing a cognitive–motivational–emotional perspective that delves into specific appraisal processes leading to distinct emotions (Smith and Lazarus, 1990).

The transactional model of stress posits that stress is determined by cognitive appraisal, which is the process by which people evaluate their interactions with the environment to comprehend the implications for their overall well-being. Accordingly, stress is a major risk factor for mental health problems (Obbarius, et al., 2021). Stress is therefore defined as: “an individual's evaluation of a relationship between themselves and their environment that is detrimental to their well-being and in which their resources are depleted or exceeded" (Folkman and Lazarus 1985, p. 152). Shiel [21] explains stress as a physical, mental, or emotional factor that induces bodily or mental tension. During an infectious disease outbreak, stress can provoke anxiety and concerns about one's health, exacerbate existing chronic health conditions, and lead to increased substance use [22]. Stressful life events, such as those triggered by the COVID-19 pandemic, significantly impact an individual's emotional and physical well-being. Furthermore, they may serve as precipitants for psychological issues, including anxiety, depressive symptoms, and others [23].

The COVID-19 pandemic has resulted in a notable increase in anxiety, with uncertainties surrounding its specific impact and risk factors across various populations [24, 25]. Anxiety disorders are the most prevalent form of mental health conditions and can be characterized by abnormally heightened levels of worry or fear. Anxiety has been linked to an elevated risk of ulcers, back pain, migraines, and asthma [26]. In extreme cases, it serves as a significant risk factor for cardiac diseases [27].

Depression, as defined by the American Psychiatric Association, entails feelings of sadness and/or a loss of interest in activities that were once enjoyable. It can lead to a range of emotional and physical problems, impairing an individual's functioning at work or home. Diagnosis typically requires the presence of symptoms for at least two weeks [28]. Clinical manifestations of depression, as highlighted by Wang, Wang & Yang [29], include a dull appearance, loss of appetite, weight loss, diminished interest, irritability, and more. Some individuals experience an excessive magnification of the disease's risks, leading to a loss of faith in treatment and society. In extreme cases, individuals may even compose suicide notes or engage in suicidal behavior.

### COVID-19 psychological pressures & depression

While numerous studies have investigated the pressure, stress, and anxiety experienced by university students (young adults) due to COVID-19 and their relationship with depression, only a limited number have examined the psychological distress faced by individuals during the COVID-19 outbreak in China, despite it being one of the most affected countries by the consequences of COVID-19. These pressures encompass various aspects related to COVID-19, including hazards and contamination (e.g., Audet et al., [15]), socioeconomic consequences [30], xenophobia [30], traumatic stress [31, 32], and compulsions [33].

Previous evidence has indicated a significant association between the psychological pressure of COVID-19 and depression, particularly in large-scale studies. For instance, Ma et al. [34] reported depressive symptoms in 21.1% of their sample of 746,217 participants in China, Wathelet et al. [35] observed depressive symptoms in 24.7% of Chinese university students (69,054 participants) due to COVID-19, and Li et al. [36] identified depressive symptoms in around 39% of their total sample of 706,415 participants affected by COVID-19. Liu et al. [37] reported depression rates ranging from 33.87% to 40.08% among 13,462 college students, while Dai & Yu [38] observed depressive symptoms in 28.93% of participants affected by COVID-19 pressures. Additionally, Li et al. [39] found that 35.2% of participants experienced depression due to COVID-19, Zhan et al. [40] reported a depression rate of 43.77%, and Park et al. [41] demonstrated a significant association between levels of depression and anxiety with COVID-19-related stress. Furthermore, Liu et al. [42] found an increase in depression and anxiety among new college students over the course of 16 months of the COVID-19 pandemic. These findings highlight the close relationship between depression and symptoms associated with the COVID-19 pandemic. Therefore, the current study hypothesizes that:

#### Hypothesis 1 COVID-19 psychological pressures are directly associated with depression among university students

##### COVID-19 psychological pressures & FOMO

Recent research has revealed that the fear of missing out (FoMO) partly contributes to high social media usage and signs of addiction among individuals [43]. FoMO refers to the pervasive fear that one may be missing out on rewarding experiences others are having. It is characterized by a constant desire to stay connected to what others are doing [44]. Participation in social media platforms tends to be particularly appealing to individuals with a fear of missing out [45] (p. 1841).

In today's digital age, people often perceive others' lives as better and filled with happier moments, especially through social media. The absence of holiday and party photos during the quarantine has further intensified individuals' fear of missing out. Consequently, digital information has significantly influenced our emotional and physical experiences during the pandemic, necessitating the development of solutions for future crises [46].

While prior studies have examined FoMO as a trait variable [45], more recent research has focused on situational cues, such as reviewing social media posts, that can trigger FoMO in specific moments [47, 48]. However, literature on the association between COVID-19 anxiety, stress, depression, and fear of missing out (FoMO) is scarce, both within and outside China. To the best of our knowledge, only a few studies have investigated this association [49–51]. Existing literature has primarily focused on the relationship between problematic mobile phone use and mental health issues, with FoMO serving as a means of escapism (e.g. [52–55],). It is logical to infer that the lockdown and home quarantine measures during the COVID-19 outbreak trigger fear among individuals, coupled with a fear of missing out on essential information for staying safe. Therefore, it is reasonable to assume a direct association between COVID-19 psychological pressures and FoMO among Chinese university students during the pandemic. Interestingly, the relationship between COVID-19 pressures and the fear of missing out (FoMO) has not been thoroughly investigated. Therefore, the current study hypothesizes that:

#### Hypothesis 2 COVID-19 psychological pressures are directly associated with FOMO among university students

##### Online Social Support as a Mediator

External social support serves as a significant indicator of depression and plays a crucial role in individuals' psychological well-being [56]. The social provisions theory posits that individuals seek specific social support functions through various relationships, and different types of relationships fulfill distinct social needs [57]. While the efficacy of social support in buffering stress remains controversial, it is well-established that social support acts as a proactive coping mechanism for stress regulation [58]. Internet platforms offer opportunities for individuals to access social support from various sources [59, 60].

College students who possess substantial online social support are more likely to utilize the Internet [59]. Zhao et al. [61] discovered that Internet usage significantly decreased COVID-19-related stress and anxiety while concurrently increasing perceived social support. Moreover, online social support was effective in fostering public confidence in overcoming the COVID-19 epidemic by influencing both cognition and emotion [62]. For university students, online social support, particularly instrumental social support, is closely linked to academic procrastination during the pandemic [63].

In terms of the mediating role of online social support in the relationship between COVID-19 psychological distress (anxiety, stress, and fear), depression, and FoMO, there is a dearth of literature addressing this gap. Only a handful of studies have examined COVID-19 stress, FoMO, problematic smartphone use, and mental health issues (depression, anxiety, stress) among college students. Gong et al. [64] found a positive association between perceived COVID-19-related strain, FoMO, problematic smartphone use, and mental health issues (depression, anxiety, stress) among college students. They also identified that FoMO partially or fully moderated the link between perceived COVID-19-related constraints and problematic smartphone use as well as mental health issues. Additionally, resilience and social support reduced FoMO, problematic smartphone use, and mental health issues (depression, anxiety, stress). Further research exploring the moderating role of online social support is warranted to delve deeper into the actual impact of online social support on the social and emotional dimensions of COVID-19-related psychological pressures and strains.

#### Hypothesis 3 Online social support will mediate the relationship between (a) COVID-19 pressures and depression and (b) COVID-19 pressures and Fear of missing out (FoMO) among university students.

##### Emotion regulation as a Mediator

Emotion regulation encompasses the processes of initiating, maintaining, and modifying one's emotional experience and expression [65, 66]. Brockman et al. [67] posit that emotion regulation strategies are not inherently good or bad. Instead, their adaptiveness depends on their appropriateness for the situation and their alignment with an individual's personal goals, rather than strict adherence. However, certain emotion regulation strategies have consistently been associated with better coping and mental health outcomes. These strategies typically involve action orientation (such as planning and problem-solving) or emotional awareness and accurate appraisal of the situation (including emotional awareness, clarity, and reappraisal) [68].

The COVID-19 pandemic has left individuals in a state of chaos. Utilizing emotion regulation strategies can provide a sense of control amidst this chaos [69]. Depressive symptoms related to the COVID-19 pandemic have been linked to the underutilization of emotion regulation strategies. Joormann & Gotlib [70] found that depression is associated with difficulties in cognitive control. Even in children, parental reports of depression symptoms were associated with poorer emotion regulation [71]. Similarly, employing effective emotion regulation strategies can help alleviate the fear of missing out [72]. Therefore, emotional regulation can play a partial moderating role in the relationship between COVID-19 pressures, depression, and the fear of missing out (FoMO) among university students.

*Hypothesis 4 Emotional regulation will mediate the relationship between (a) COVID-19 pressures and depression and (b) COVID-19 pressures and Fear of missing out (FoMO) among university students.*

## Methods

### Research design

A descriptive research design using an online questionnaire was employed to gather data from Chinese university students.

### Samples and settings

Data collection for the study lasts for 4-month periods as it took place between July 22nd, 2020, and November 2nd, 2020 during the peak of Covid-19 outbreak. The data in this study were collected during the peak of the COVID-19 outbreak, specifically the resurgence of the Delta variant. The study comprised 521 full-time university students currently enrolled in Chinese universities. Of the participants, 81.0% identified as female, and nearly half of the sample (56.0%) were undergraduate students. The vast majority of respondents (91.7%) reported not currently suffering from any illness. Initially, approximately 650 university students were invited to participate in the study, and over the course of the 4-month data collection period, 521 students responded, resulting in a response rate of 80.15%.

### Instruments

COVID-19 pressures were assessed using the COVID Stress Scales developed by Taylor et al. [73]. The scale consisted of 36 items designed to measure various aspects of COVID-19-related stress. The COVID Stress Scales encompassed five subscales: COVID-19-related danger and contamination (e.g., "*I am worried about catching the virus*" and "*I am concerned about the possibility of getting infected by touching public surfaces such as handrails or door handles*"), COVID-19-related socio-economic consequences (e.g., "*I am worried about shortages of prescription medicines at pharmacies*"), COVID-19-related xenophobia (e.g., "*I would feel concerned if I encountered someone from a foreign country due to the possibility of them having the virus*"), COVID-19-related traumatic stress (e.g., "*I experienced unwanted, disturbing mental images related to the virus*"), and COVID-19-related compulsive behaviors (e.g., "*I frequently sought advice from healthcare professionals such as doctors or pharmacists regarding COVID-19*").

The scale items inquire about various concerns participants may have had during the past week regarding the infection. Participants rated items on a 5-point Lickert scale ranging from 1 (not at all) to 5 (extremely). The term 'worries' was used to assess feared (anticipated) outcomes. For the checking and traumatic stress items, the rating scale based on 5-point Lickert scale ranged from 1 (*never*) to 5 (*almost always*). The COVID Stress Scales demonstrated good internal consistency, with Cronbach's alpha coefficients ranging from .84 to .91 for the subscales. Additionally, the scale exhibited high convergent validity with other measures of anxiety and stress.

Depression was assessed using the Depression, Anxiety, and Stress Scale - 21 Items (DASS-21) developed by Lovibond and Lovibond [74] and Henry and Crawford [75]. The DASS-21 consists of 21 items and is composed of three self-report scales designed to measure depression, anxiety, and tension/stress. Each of the scales contains 7 items, which are further divided into subscales with comparable content. Participants rated each item on a 4-point Likert scale ranging from 0 (*Did not apply to me at all*) to 3 (*Applied to me very much, or most of the time*). Previous studies consistently demonstrate excellent psychometric properties of the DASS-21, with internal consistency values ranging from 0.83 to 0.94 [75–78].

Fear of Missing Out (FoMO) was assessed using The Fear of Missing Out (FoMO) scale developed by Przybylski et al. [45]. The FoMO scale is a 10-item self-report tool that measures fear of missing out across four factors: missed experiences, compulsion, comparison with friends, and being left out. Example items include "*I fear others have more rewarding experiences than me*" and "*When I go on vacation, I continue to keep tabs on what my friends are doing*". Participants rated each item on a 5-point Likert scale (1 = *disagree*, 5 = *agree*). Previous studies consistently report excellent psychometric properties of the FoMO scale, with internal consistency values of α = .87 to .90 [45] and .90 [79].

Online social support was assessed using an adapted version of the Online Social Support for Smoker Scale [80], modified to be Covid-19 oriented. The scale comprises 15 items that primarily assess the social support individuals receive through social media networks. Example items include "*I connected with other people on social networks on topics other than Covid-19*" and "*using social networks helped me cope with Covid-19 precautionary procedures*" and "*advice and support from people on common interest groups and forums about the Covid-19 pandemic are really important*". Participants rated each item on a 4-point Likert scale ranging from 1 (*disagree a lot*) to 4 (*agree a lot*). The Covid-19 Online Social Support Scale demonstrated good internal consistency in this study, with a Cronbach's alpha coefficient of .89 for the total scores.

Emotional regulation was assessed using the Emotion Regulation Questionnaire (ERQ) developed by Gross and John [81], based on Gross's [82] process model of emotion regulation. The ERQ is a 10-item self-report questionnaire that measures individuals' use of two emotion regulation strategies: cognitive reappraisal and expressive suppression. Cognitive reappraisal involves changing how one thinks about a situation to alter its emotional impact, while expressive suppression involves inhibiting the expression of emotions. Participants rated each item on a 7-point Likert scale, ranging from 1 (*strongly disagree*) to 7 (*strongly agree*), with higher scores indicating greater use of emotion regulation strategies [81, 83]. The emotional regulation questionnaire demonstrated good internal consistency in this study, with a Cronbach's alpha coefficient of .79 for the total scores.

### Data collection and ethical clearance

The study received ethical approval from the Research Review Committee of the university. Questionnaires were administered to participants through an online platform, Google Forms, delivered via their email addresses and social media accounts. The completion time for the questionnaires ranged from 20 to 30 minutes. Prior to completing the survey, participants were provided with information about the study and informed of their voluntary participation, with the option to withdraw at any time. The cover sheet of the online questionnaire outlined the study's purpose, as well as the associated risks and benefits. Following the cover sheet, a brief letter requested participants' consent. Participants who agreed to participate in the study were instructed to click the "*Continue*" button, while those who chose not to participate were directed to press the "*stop*" button. Weekly reminder emails were sent to participants throughout the study period.

### Data analysis

Obtained data has been analyzed using the Statistical Package for Social Sciences (SPSS) 26.0. At the very beginning of the data analysis, descriptive statistics including frequencies and percentages have been calculated. Bivariate analyses were conducted using analysis of variance, t-tests, and Pearson's correlation coefficient to examine the relationships between variables. To investigate the direct and indirect effects of COVID-19 pressure on university students' experience of depression and fear of missing out (FoMO), a multilevel regression analysis was performed. In the first step, the independent variable, psychological pressure from COVID-19, was regressed on the mediators, online social support, and emotional abilities. Next, COVID-19 psychological pressure was regressed on the dependent variables, depression, and FoMO in university students. Finally, the mediators regressed on the outcome variables while controlling for the independent variables.

Selecting the cut-off values for scales used in the current study is crucial, balancing sensitivity and specificity. In order to decide the optimal cut-off values, the authors used ROC curve analysis which involves data collection, sensitivity/specificity calculation, ROC curve plotting, optimal cut-off determination, Youden's Index, context and goal consideration, and validation on an independent dataset. The authors acknowledge the variability of optimal cut-offs based on population, context, and study goals, underscoring the importance of careful consideration by researchers.

## Results

Almost all participants reported not currently experiencing any illnesses at the time of their participation in the study (91.7%) (see Table 1).

**Table 1:** Participants’ characteristics

Characteristics	Categories	N.	%
Age (18-43)			
Gender	Male	99	19.0
	Female	422	81.0
Study level	undergraduates	292	56.0
	postgraduates	229	44.0
Socio-economic level	Below average	14	2.7
	Average	410	78.7
	Above average	97	16.6
Current illness	Yes	43	8.3
	No	478	91.7

Bivariate tests were conducted to examine the relationships between key study variables, as presented in Table 2. Consistent with expectations, Covid-19 psychological pressures were found to have significant positive correlations with depression (*r* = .339, *p* < .001), fear of missing out (FoMO) (*r* = .236, *p* < .001), and emotion regulation (*r* = .330, *p* < .001). Furthermore, Covid-19 psychological pressures showed a significant negative correlation with online social support (*r* = - .533, *p* < .001). However, no significant correlations were found between students' characteristics and Covid-19 psychological pressures, depression, and FoMO.

**Table 2:** Correlations between key study variables

Variables	Mean	SD	1	2	3	4	**5**
1. Covid-19 psychological pressures	99.14	24.76	-				
2. Depression	16.08	12.05	.339^**^	-			
3. FOMO	26.62	7.36	.236^**^	.400^**^	-		
4. Online Social Support	26.22	9.02	-.533^**^	-.223^**^	-.234^**^	-	
5. Emotion regulation	43.23	7.85	-.330^**^	-.507^**^	.451^**^	.216^**^	-

Three-stage regression analyses were conducted to examine the direct and indirect effects of COVID-19 psychological pressures on students' outcomes, as presented in Table 3. The results revealed that COVID-19 psychological pressures had significant direct positive effects on depression (*β* = .165, *p* < .001), fear of missing out (FoMO) (*β* = .072, *p* < .001), and negative effects on emotion regulation (*β* = -.371, *p* < .001). Online social support showed significant direct negative effects on depression (*β* = -.298, *p* < .001) and fear of missing out (FoMO) (*β* = -.195, *p* < .001).

**Table 3:**
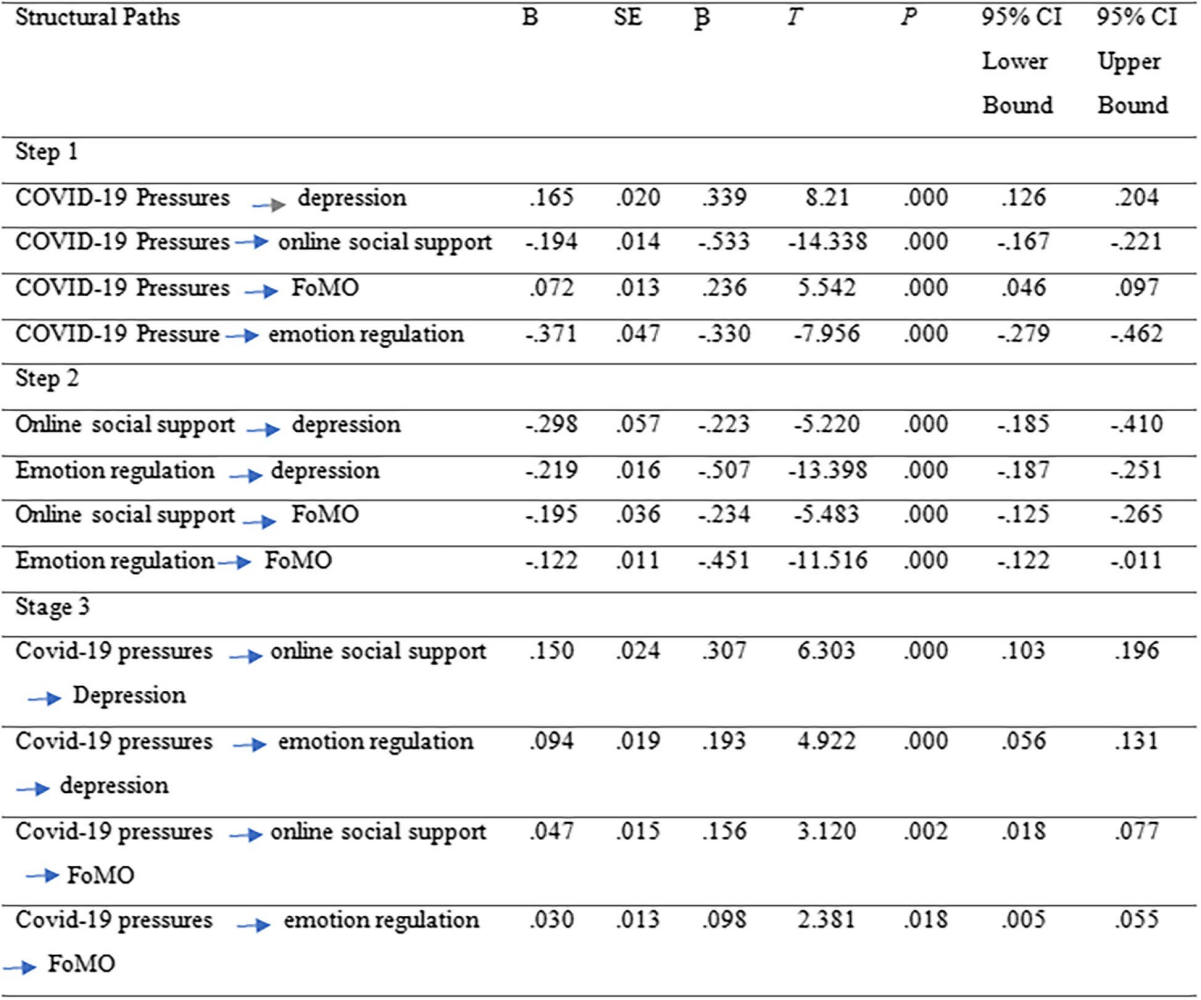
Direct and indirect effects estimates

Furthermore, through the mediating role of online social support, COVID-19 psychological pressures had significant indirect effects on depression (*β* = .150, *p* < .001) and fear of missing out (FoMO) (*β* = .047, *p* < .001). Additionally, emotion regulation partially mediated the association between COVID-19 psychological pressures and depression (*β* = .094, *p* < .001), as well as between COVID-19 psychological pressures and fear of missing out (FoMO) (*β* = .030, *p* < .018).

## Discussion

Based on a cut-off score of >99, a total of 287 university students (55.09%) were found to experience COVID-19 psychological pressures, including fear of danger and contamination, concerns related to socio-economic consequences, xenophobia, traumatic and post-traumatic issues, and compulsive checking for online assistance regarding the virus outbreak. This study aims to investigate the association between the target variables, particularly fear of missing out (FoMO) and emotional regulation, in relation to COVID-19 psychological pressures among Chinese university students during the pandemic. This study aligns with various reports that have examined the presence of coronavirus-related psychological pressures in university students since the outbreak began [84, 85]. However, the prevalence of COVID-19 psychological pressures and stresses in the study sample was higher than in studies involving other college students [86].

The participants in this study reported difficulties in overcoming the psychological pressures experienced during the pandemic, as they had no clear understanding of when the pandemic would end and lacked confidence in the efficacy of available treatments (at that time) to protect them from the viral infection. Notably, the study's findings revealed a significant proportion of Chinese university students (43.57%, *n*=227) expressing fear of direct contact with foreigners, which is a unique aspect addressed in this study. While numerous studies have investigated xenophobia and stigmatization, few have explored Chinese individuals' fear of getting infected due to close contact with foreigners or non-Chinese individuals.

Previous studies examining xenophobic behaviors during the pandemic have shown illogical and unexplainable stigmatization not only towards Chinese individuals but also towards Asians in general [87]. It should be noted that xenophobic attitudes among Chinese individuals during COVID-19 were primarily limited to the fear of physical contact with foreigners and did not extend to online bullying practices. This finding contradicts studies conducted during the pandemic, such as the detrimental effects of xenophobic bullying and propaganda on the physical and mental health of Asian Americans in the United States [88–90] and the abandonment and boycott of Chinese companies and communities in Asian ethnic enclaves [91]. The increasing reliance on technology and the proliferation of misinformation, pseudoscience, and xenophobic ideologies may have contributed to these disparities [92].

Regarding the prevalence of fear of contamination during COVID-19, 34.54% (*n*=180) of participants reported high anxiety regarding viral contamination through contact with surfaces and objects. Concerns have been raised about individuals with obsessive-compulsive disorder (OCD) experiencing increased fear of contagion/contamination during the COVID-19 pandemic, particularly as the severity of the pandemic intensified [15].

Furthermore, 20.54% of students reported excessive reliance on web surfing to obtain COVID-19-related information, compared to 13.97% reporting severe cyberchondria. This behavior manifested as continuous checking of online health platforms and social media networks. It is important to note that the data in this study were collected during the peak of the COVID-19 outbreak, specifically the resurgence of the Delta variant. China established hotlines to respond to inquiries, yet individuals continued to rely heavily on websites for COVID-19-related health information. The limitations imposed by the virus transmission hindered face-to-face interactions, including social interactions and psychological services. Consequently, China witnessed a surge in the utilization of various online mental health services [93]. However, experiences in China have shown that online mental health services cannot replace crisis hotlines. Prior to the pandemic, China had 63 crisis hotlines, but as of March 27, 2020, over 200,000 calls had been answered by 625 hotlines in 31 Chinese provinces [94].

### Effects of independent variables on mediators

During the pandemic, the combination of COVID-19 psychological pressures and high levels of depressive symptoms was associated with fear of missing out (FoMO) among university students (*r* = .236, *p* < .001) due to the lockdown measures. The COVID-19 pandemic significantly reduced opportunities for social encounters, leading to increased reliance on social media for maintaining social relationships. The limited scope of social activities and friendships during the quarantine period necessitated an investigation of the fear of missing out (FoMO) [95]. The fear of missing out has been found to be strongly associated with social media usage, particularly among younger individuals [96]. However, the pandemic posed greater concerns and potential impacts on students compared to previous circumstances [97].

As schools and universities implemented lockdown measures, social media usage driven by the fear of missing out increased. Distance learning became the primary approach for higher education institutions to mitigate the negative impact of disrupted classes [98]. In this study, students who experienced psychological pressures, depressive symptoms, stress, and anxiety due to virus infection were found to have higher levels of fear of missing out (FoMO) and a tendency to rely excessively on online sources to stay informed about the pandemic.

The implementation of precautionary confinement resulted in limited socialization and engagement with others within the community, leading to social and emotional isolation. The persistent implementation of social distancing protocols potentially exacerbated the psychological difficulties experienced by students, contributing to increased feelings of loneliness and isolation (Bu, Steptoe & Fancourt, 2020). Several studies focusing on university students have reported a notable increase in the proportion of students facing psychological pressures since the pandemic's onset. This increase can be attributed primarily to social distancing measures and lockdown protocols implemented worldwide to contain the virus's spread [8, 40].

For young adults, social interaction is crucial for identity formation and social-emotional well-being [99]. Therefore, it is important to provide measures that foster social connectedness through innovative means. The current study found a direct and positive association between COVID-19 psychological pressures and fear of missing out (FoMO) among university students, which aligns with previous limited evidence linking coronavirus-induced anxiety to a higher occurrence of fear of missing out behaviors during the pandemic among university students [97]. Given the scarcity of literature on the association between COVID-19 stress and fear of missing out (FoMO), this study contributes to filling a significant gap in the literature.

### Effects of independent variables on outcomes

The direct effect of COVID-19 psychological pressures on depressive symptoms observed in students during the pandemic highlights the importance of implementing social support interventions to reduce depression among students during the coronavirus outbreak and subsequently alleviate fear of missing out (FoMO). One key finding of this study is the partial mediating effect of online social support on the relationship between COVID-19 psychological pressures and depression, as well as between COVID-19 psychological pressures and fear of missing out (FoMO). In other words, the negative effects of COVID-19 psychological pressures on students' well-being and mental health were reduced when adequate online social support was provided.

Findings of this study indicate a direct correlation between stress and individuals' mental well-being. Several theoretical frameworks suggest that being exposed to stressors, such as those caused by the COVID-19 pandemic, can have a substantial effect on mental health outcomes. The Lazarus and Folkman (1984) transactional model of stress and coping is frequently utilized to comprehend how individuals perceive and react to stressors. Amidst the pandemic, Chinese students may experience intensified psychological pressures, which can be seen as stressors that contribute to a rise in depression symptoms.

Furthermore, the study underscores the crucial need of leveraging online social support as a primary intervention to mitigate the adverse effects of psychological stressors induced by COVID-19. The availability of online counselling services, virtual support groups, and community forums has become essential in enabling students to interact with others and receive emotional support (Browning, et al. 2023; Fini, Tummolini & Borghi, 2021; Kenyon, Kinakh & Harrison, 2023; Kühne, Fischer & Jeglinski-Mende, 2022; Monninger, 2023; Sahi, et al. 2021). These online platforms offer a safe and convenient environment for students to exchange their experiences, seek guidance, and gain reinforcement from those who may be facing similar difficulties. Furthermore, the affordability of online social support empowers students to engage in self-help techniques and nurture coping mechanisms that might home their psychological suffering amongst these uncertain times.

One key finding of this study is the partial mediating effect of online social support on the relationship between COVID-19 psychological pressures and fear of missing out (FoMO). Individuals who experienced higher levels of COVID-19 psychological pressures were more likely to report higher levels of fear of missing out. However, the presence of online social support partially mediated this relationship, suggesting that having access to supportive online networks can help mitigate the negative impact of COVID-19 psychological pressures on FoMO (Dempsey, et al., 2019; Gong, et al., 2022; Marciano, et al., 2022; Tandon, et al., 2021). This highlights the need of receiving online social support to mitigate the adverse impacts of psychological pressures triggered by COVID-19 on the fear of missing out. It indicates that those who have access to supportive online networks are more likely to effectively handle the obstacles presented by the pandemic and maintain a healthier level of Fear of Missing Out (FoMO).

In terms of practical implementation, instructional adaptations are crucial for addressing students' mental health in educational institutions. Practical strategies to support students during the epidemic include incorporating mental health resources into remote learning platforms, coordinating virtual wellness sessions, and lobbying for mental health awareness campaigns. These adjustments can facilitate equitable access to essential resources and support for students, irrespective of their geographical location. In addition, educational institutions can engage in collaboration with mental health specialists and organizations to formulate comprehensive solutions that effectively address the distinctive difficulties encountered by students in these current circumstances (Al-Kumaim, et al., 2021; Dayagbil, et al., 2021; Leal Filho, et al. 2021; Singh, Steele & Singh, 2021; Wiedermann, et al., 2023).

Moreover, establishing a sense of camaraderie, even in a virtual setting, is crucial for students grappling with the isolating consequences of the pandemic. Practical applications involve creating virtual platforms where students can exchange experiences, participate in mutual assistance, and maintain a sense of connection despite the adoption of physical distancing measures. These digital platforms encompass virtual clubs, discussion forums, and social media groups where students can engage and provide mutual assistance. In addition, educational institutions have the ability to arrange virtual events and activities that foster a feeling of inclusion and motivate students to actively engage in the online community (Hollister, et al., 2022; Nandlall, et al., 2022; Sage, et al., 2021; Tang, et al., 2023).

### The effects of the mediators on outcomes

Results also suggest that when students have access to desirable online social support, they can effectively manage the impact of the pandemic, leading to reduced depressive symptoms, fear of missing out (FoMO), and improved emotion regulation. Social support, which involves sharing resources through relationships, has been extended to the online context, known as online social support [100], and has been consistently associated with positive outcomes for mental and emotional health [62]. Research conducted during the COVID-19 pandemic has demonstrated that adequate online social support is strongly linked to reduced anxiety, stress, depression, and loneliness among college students, contributing to overall improvements in individuals; mental health [101–103]. Conversely, the absence of online or offline social support in young adults has been associated with heightened mental and psychological distress, especially during pandemics [104, 105].

Effective utilization of adaptive emotional regulation mechanisms, such as reappraisal, has been shown to reduce stress-related feelings and physical illnesses. In contrast, dysfunctional emotional regulation strategies like rumination and emotion suppression are associated with the etiology of depression and physiological diseases [106]. The COVID-19 outbreak placed individuals in a state of chaos, facing various challenges that adversely affected their mental health and well-being [69]. These challenges disrupted essential aspects of life, including work, family responsibilities, and daily activities [107]. According to Restubog, Ocampo, & Wang [69], effective emotion regulation can mitigate the destructive and disruptive impact of COVID-19-related stressors.

Interestingly, no previous study has investigated how emotion regulation can help reduce fear of missing out (FoMO), making this aspect worth exploring. After an extensive literature review, we found only one poster [108] that attempts to establish a framework for the impact of emotion regulation on reducing FoMO. Therefore, the results obtained in this study, regarding the partial mediation of emotion regulation in the relationship between COVID-19 psychological distress and fear of missing out (FoMO), hold significant importance. Based on our expectations, effective use of emotion regulation strategies is negatively associated with higher levels of psychological distress, depression, and fear of missing out (FoMO). During the pandemic, the combination of lockdown measures and psychological stress has heightened the need for internet-based emotional intervention programs that promote better emotion regulation among university students, leading to reduced depression and improved well-being.

Lastly, online social support was found to partially mediate the association between COVID-19 psychological pressures and depression, as well as between COVID-19 psychological pressures and fear of missing out (FoMO) among university students. This finding suggests that when students are provided with adequate online social support, the negative consequences of psychological distress, depressive symptoms, and fear of missing out (FoMO) can be mitigated, resulting in reduced depression and less fear of missing out (FoMO). Previous research conducted before the pandemic consistently highlighted online social support derived from friends, peers, or communities as a critical precursor to improved emotional, mental, and psychological health across various age groups [8, 109].

### The mediating effects

The partial mediating effects of social support and emotional skills, such as reduced alexithymia leading to increased emotional competence, on the association between COVID-19 mental distress and depression, as well as fear of missing out (FoMO) among university students, suggest that other factors should be considered in future investigations. Personal factors, such as a sense of coherence, optimism, resilience, self-efficacy, self-care skills, and personal health and well-being, could potentially mediate the influence on university student outcomes. Moreover, it is crucial to investigate the impact of contextual variables, such social media use, academic stress, along with the accessibility of mental health services, in comprehending the correlation between mental health issues caused by COVID-19 and depression. Future study can enhance the knowledge of the intricate relationship between different variables and their influence on university student outcomes during crises by taking into consideration these supplementary aspects (Kupferberg & Hasler, 2023; Wang, et al., 2023; Zhang, et al., 2023).

Additionally, the level of social interaction, social skills, and social connection or contact may also contribute to understanding the mechanism underlying the relationship between COVID-19 psychological pressures and depression, as well as fear of missing out (FoMO) among university students. These factors should be taken into account in future research endeavors. Moreover, evaluating the influence of these elements has the potential to result in the development of efficient interventions and support systems for college students. Gaining insight into the impact of psychological stressors related to COVID-19 on the academic performance and well-being of college students is essential in order to devise efficient treatments and support mechanisms. Future research should investigate the impact of academic stress and financial issues on the exacerbation of depression and fear of missing out (FoMO) among university students, in addition to examining the influence of social contact and social skills (Barbayannis, et al., 2022; Copeland, et al., 2022; Córdova Olivera, et al., 2023; Kokkinos, Tsouloupas & Voulgaridou, 2022; Rahiman, et al., 2023; Tang & He, 2023). Through a thorough analysis of these elements, we can more effectively tackle the mental health difficulties experienced by students amidst this pandemic and equip them with the essential resources for achieving success.

## Conclusion

The findings of this study highlight the prevalence of COVID-19 psychological pressures among university students and young adults during the pandemic. It was observed that students who experienced COVID-19 psychological pressures and stress were more likely to report symptoms of depression and an increased occurrence of fear of missing out (FoMO). The co-occurrence of COVID-19 pressures, depressive symptoms, and high FoMO levels also exacerbated students' alexithymia, or the difficulty in expressing emotions. However, the provision of adequate online social support significantly mitigated the negative consequences of COVID-19 psychological pressures among university students, leading to reduced levels of depression and FoMO.

In response to these findings, it is crucial for college administrations and teaching staff to develop measures and strategies aimed at enhancing online social support for students in higher education institutions. By incorporating these measures into organizational strategies, student well-being can be enhanced, depression can be minimized, and the detrimental effects of FoMO and alexithymia can be alleviated, ultimately fostering academic success during the pandemic. Given the prevailing limitations imposed by the COVID-19 pandemic, it is essential to explore the potential of technology and web-based approaches in maintaining social connectedness and delivering online social support interventions.

## Limitations and future research

An important limitation of the present investigation is the possibility that the results may have restricted applicability to a broader population due to the sampling methodology utilized. One potential explanation for the underrepresentation of certain individuals in the sample could be their lack of internet access or reluctance to partake in online surveys. Furthermore, it is imperative to investigate perplexing variables associated with the unique attributes of the sample population, including socioeconomic and cultural factors. To augment the external validity of the results, subsequent inquiries ought to strive to ascertain cohorts that are more diverse and representative.

An additional constraint pertains to gender bias and the representativeness of the sample. Due to the preponderance of female participants, the representativeness of the sample may have been compromised, and gender-related biases could have been introduced into the findings. In an effort to rectify this constraint, forthcoming investigations ought to endeavour for a more equitable portrayal of gender. This would facilitate a more comprehensive comprehension of the potential variations in the relationships investigated in the study among distinct gender cohorts, thereby enhancing the nuanced interpretation of the results.

Besides, utilizing self-report instruments for the purpose of data collection may represent another limitation of this study. This introduces the potential for bias due to a single method. Social desirability or subjective interpretations may impact the responses of participants, resulting in an inadequate comprehension of the psychological constructs being studied. Further investigation is warranted to explore the integration of various measurement techniques, including physiological and behavioural observations, to attain a more exhaustive and unbiased evaluation of the variables under investigation.

The research suggests that future studies should consider longitudinal investigations to understand the impact of the pandemic on university students' mental health outcomes, emotional regulation, psychological pressures, and online social support. This would allow for a more nuanced understanding of the pandemic's effects on students and provide insights into cultural differences in response to stressors. Cross-cultural studies and comparative analyses within diverse cultural environments could also provide valuable insights into how different cultures react to stressors. Mixed-methods approaches, combining qualitative and quantitative methods, could enhance the reliability and validity of the results. The study also suggests that future research should examine the efficacy of targeted interventions addressing online social support and emotional modulation to alleviate the adverse effects of psychological pressures associated with COVID-19. This could lead to the development of evidence-based interventions promoting the mental health and overall well-being of students who face similar challenges. By acknowledging these limitations and exploring future research avenues, we can better understand the complex relationship between psychological pressures, emotion regulation, online social support, and mental well-being among college students.

## Data Availability

The data supporting the findings of this study can be obtained from the corresponding author upon reasonable request.
